# Allometric growth rate of the spinal cord in relation to the vertebral column during prenatal life in male and female goats (*Capra hircus*)

**Published:** 2016

**Authors:** S. M. Ghazi, R. Ranjbar, M. Khaksary Mahabady

**Affiliations:** 1 Ph.D. Student in Comparative Anatomy and Embryology, Department of Anatomical Sciences, Faculty of Veterinary Medicine, Shahid Chamran University of Ahvaz, Ahvaz, Iran;; 2 Department of Anatomical Sciences, Faculty of Veterinary Medicine, Shahid Chamran University of Ahvaz, Ahvaz, Iran

**Keywords:** Allometric growth rate, Goat fetus, Termination of the spinal cord

## Abstract

Total and regional allometric growth rates and termination sites of the spinal cord related to the respective vertebra were studied in 36 goat fetuses, from the Ahvaz slaughterhouse. These specimens were assigned to 3 groups, group 1 (CRL 10-20 cm), group 2 (CRL 21-30 cm), and group 3 (CRL 31-40 cm), each consisting of 6 male and 6 female fetuses. Observations in all 3 groups revealed that although the growth of the vertebral column was greater than that of the spinal cord, the difference in growth was not constant throughout the spine. While in cervical and thoracic regions the growth rate of the spinal cord in relation to the vertebral column was almost isometric, in the caudal part of the spine there was marked decline in growth of the spinal cord compared to the respective regions of the vertebral column. Craniocaudally, the allometric growth rate became drastically negative. There was no significant difference (P>0.05) between males and females. Except in thoracic region, all other regions showed significant differences (P<0.01) between similar regions in all 3 groups. In the lumbar region of group 2 no significant difference was found (P>0.05). As a consequence of the negative allometric growth of the spinal cord in relation to the vertebral column in the caudal part of the spine, the conus medullaris was displaced from S4-S5 in group 1 to S2 in group 3. No significant difference (P>0.05) between male and female fetuses concerning the termination of the spinal cord was found.

## Introduction

As early as 1918, Kunitomo and later on in 1919 Streeter described that the spinal cord and the vertebral column lie side by side in a metameric manner in the early stage of development. During the short period of the early embryonic stage, the spinal cord grows at almost the same rate as the vertebral column, thus displaying isometric growth. This phase, however, is of short duration since the adjacent vertebral segments begin to increase in length more rapidly than those of the spinal cord. The growth of the spinal cord segments in relation to the respective vertebrae thus exhibits negative allometry which continues even up to adult life. The difference in the growth rate between the spinal cord and the vertebral column was an important factor in the caudalmost segments of the spinal cord to form the cauda equina and the filum terminale. Although the develop-mental patterns of the spinal cord and vertebral column have been examined independently but the results of these separate studies have not been correlated. The observations of Barry (1956) ▶, Nizankowski and Kurlej (1982) ▶, and Roth and Purkyne (1985) ▶ in man, Fletcher and Kitchell (1966) ▶, and Morgan et al. (1987) ▶ in dogs, Hifny et al. (1984) ▶ in donkeys, Sakla (1969) ▶ in the Albino mouse, revealed the neurovertebral growth relations. Ghazi and Gholami (1993a, b and 1994) in sheep, Gholami et al. (1997 ▶), and Ghazi et al. (1998) ▶ in one humped male camel, Ghazi et al. (2000d) ▶ in the male and female domestic goose, Ghazi et al. (2000a) ▶ in fowl and turkey, Ghazi et al. (2000c) ▶ in rat, Ghazi et al. (2000b) ▶ in rabbit, Ghazi et al. (2001b) ▶ in the male dog, Khaksar et al. (2002) ▶ in adult male and female pigeons, Ghazi et al. (2004a ▶, b ▶) in the male cat and Rahmanifar et al. (2008) ▶ in chick and adult male ostrich, studied allometric growth rate and the termination of the spinal cord, but so far there is no information about the allometric growth rate of the spinal cord in relation to the vertebral column in goat fetuses.

The present investigation was therefore designed to study the normal allometric growth rate of the spinal cord in relation to the vertebral column for total and regional levels during prenatal life in goat. Also the developmental changes of termination site of the spinal cord were studied.

## Materials and Methods

A total of 36 native goat fetuses of both sexes were included from Khuzestan province. The fetuses were assigned into 3 groups: 10-20 cm, 21-30 cm and 31-40 cm of crown rump length (CRL). Each group consisting of 12 animals (6 male and 6 female). The fresh fetuses were collected from the slaughterhouse. They were selected and aged according to the method of Taluja et al. (1989). ▶ All selected fetuses were skinned and eviscerated. Subsequently, the axial skeleton was separated and submerged in 10% buffered formalin to protect its normal anatomical condition. The spinal cords were exposed by laminectomy and kept in the same fixative for 1 week. The duramater was then slit along the dorsomedian sulcus. The arachnoid was either lifted off or gently pushed back from the dorsal roots. In each animal, the total and regional length of the spinal cord as well as the length of their respective vertebrae was measured. The length of each spinal cord segment (in mm) was determined by measuring the distance from the most rostral rootlet of the dorsal root of each spinal nerve back to the most rostral rootlet of the dorsal root of the adjacent next spinal nerve. The length of each vertebral segment (in mm) was determined by measuring the distance between the cranioventral end of each vertebra to the caudal periphery of successive intervertebral disc, using calipers and a ruler. The length of each region was determined by adding the lengths of the relevant spinal cord segments and respective vertebrae. The total lengths of the spinal cord and vertebral column were measured by adding the length values of all regions. The allometric growth rate (mm/mm) was calculated by dividing the length of the spinal cord by the length of its related vertebrae for its total length and the regional levels.

A longitudinal incision was then given further back in the dural sac to expose the exact position of the conus medullaris showing the termination of the spinal cord. For further observation on the termination of the spinal cord at the level of related vertebrae, the body of each vertebra was divided into cranial, middle and caudal regions.

The data were analysed statistically, using a factorial analysis of variance test and Duncan procedure.

## Results

The allometric growth rate was highest in the cervical and thoracic regions, followed by the lumbar, sacral and coccygeal regions. These differences were significantly different (P<0.01) between all regions within each group while there was no significant difference between respective regions of the male and female fetuses. The total allometric growth rate of the spinal cord in relation to the vertebral column varied according to the crown rump length at which the fetuses were examined. In the male fetuses, the total allometric growth rate in group 1 (CRL 10-20 cm) was 0.81 ± 0.01, in group 2 (CRL 21-30 cm) was 0.77 ± 0.02 and in group 3 (CRL 31-40 cm) was 0.76 ± 0.02, while in the female fetuses these values were 0.81 ± 0.01, 0.78 ± 0.02, and 0.75 ± 0.01, respectively. In the cervical and thoracic regions of animals from all age groups, the allometric growth rate was about 1, implying that during prenatal life the spinal cord and vertebral column of goat in these 2 regions grew at the same rate. In the lumbar region the growth of vertebral column was greater than that of the related part of the spinal cord, but this difference was much more pronounced in the sacral and coccygeal regions. In the coccygeal region, the allometric growth rate of the spinal cord in relation to the coccygeal vertebral column was markedly decreased. In the male fetuses in group 1 it was 0.16 ± 0.02, in group 2 was 0.11 ± 0.02 and in group 3 was 0.09 ± 0.01, while in the female fetuses these values were 0.16 ± 0.02, 0.10 ± 0.02 and 0.09 ± 0.01, respectively. The allometric growth rate of the spinal cord in relation to the vertebral column in total and all regions of both sexes were shown in Table 1.

**Table 1:** Allometric growth rate of spinal cord in relation to the vertebral column in male and female goat fetuses (mean±SD

Allometric growth rate (mm/mm) in male goat fetuses						
Crown rump length (cm)	Cervical region	Thoracic region	Lumbar region	Sacral region	Coccygeal region	Total
10-20	1.01 ± 0.01*	1.00 ± 0.00	0.89 ± 0.07*	0.64 ± 0.05*	0.16 ± 0.02*	0.81 ± 0.01*
21-30	1.06 ± 0.02*	1.01 ± 0.01	0.85 ± 0.04	0.51 ± 0.08*	0.11 ± 0.02*	0.77 ± 0.02*
31-40	1.08 ± 0.02*	1.00 ± 0.02	0.83 ± 0.05*	0.38 ± 0.02*	0.09 ± 0.01*	0.76 ± 0.02*
Allometric growth rate (mm/mm) in female goat fetuses						
10-20	1.01 ± 0.02*	1.00 ± 0.01	0.87 ± 0.06*	0.65 ± 0.06*	0.16 ± 0.02*	0.81 ± 0.01*
21-30	1.06 ± 0.04*	1.01 ± 0.02	0.83 ± 0.05	0.52 ± 0.07*	0.10 ± 0.02*	0.78 ± 0.02*
31-40	1.09 ± 0.04*	1.00 ± 0.02	0.82 ± 0.03*	0.36 ± 0.04*	0.09 ± 0.01*	0.75 ± 0.01*

* Values in columns with are significantly different (P<0.01)

**Table 2:** Termination of the spinal cord in the male and female goat fetuses

Crown rump length (cm)	Termination of spinal cord in male goat fetuses				
10-20	Cranial third of S4 2 cases (33.3%)	Middle third of S4 1 case (16.7%)	Cranial third of S5 1 case (16.7%)	Middle third of S5 1 case (16.7%)	Caudal third of S5 1 case (16.7%)
21-30	Caudal third of S2 1 case (16.7%)	Middle third of S3 1 case (16.7%)	Caudal third of S3 2 cases (33.3%)	Cranial third of S4 2 cases (33.3%)	
31-40	Cranial third of S2 4 cases (66.7%)	Caudal third of S2 2 cases (33.3%)			
Crown rump length (cm)	Termination of spinal cord in female goat fetuses				
10-20	Cranial third of S4 3 cases (50%)	Cranial third of S5 2 cases (33.3%)	Caudal third of S5 1 case (16.7%)		
21-30	Caudal third of S2 1 case (16.7%)	Cranial third of S3 3 cases (50%)	Caudal third of S3 1 case (16.7%)	Cranial third of S4 1 case (16.7%)	
31-40	Cranial third of S2 5 cases (83.3%)	Caudal third of S2 1 case (16.7%)			

As a consequence of the negative allometric growth of the spinal cord in relation to the vertebral column in the caudal regions of the spine, the spinal cord terminated at the variable distance cranially according to the age of the fetuses. The anatomical levels of the caudal tip location of the conus medullaris are shown in Table 2 at different ages.

## Discussion

The anatomical relationship of the spinal cord and vertebral column in various animals is very important for understanding both the phylogenetic relationships and the functioning of the spinal cord. For obtaining more objective picture of the anatomical relationships, these studies must be complemented by quantitative analysis.

The total allometric growth rate in goat fetuses varied according to the age. It was decreased from group 1, (0.81 ± 0.01) to group 3 (0.76 ± 0.02). There was no significant difference in allometric growth rate according to the sex of fetuses. The total allometric growth rate were 0.74 ± 0.00 in new born one humped camel (Ghazi et al., 1998 ▶), 0.83 ± 0.01 in new born lamb (Ghazi and Gholami, 1994 ▶), 0.60 ± 0.02 in new born dog (Ghazi et al., 2001b ▶), 0.74 ± 0.02 in new born rabbit, 0.78 ± 0.02 in new born guinea pig and 0.66 ± 0.01 in new born rat (Ghazi et al., 2001a ▶) and 1.00 ± 0.01 in chick ostrich (Rahmanifar et al., 2008 ▶).

The present study has shown that although the vertebral column grows faster than the spinal cord, this is not true for all regions. The allometric growth rate of the spinal cord in relation to the vertebral column of the goat fetuses in the cervical and thoracic regions was about 1.00 in all age groups, while this value for the caudal part of spine was decreased drastically. For example in the group 1 it was 0.89 ± 0.07, 0.64 ± 0.05, and 0.16 ± 0.02 for lumbar, sacral and coccygeal regions, respectively.

These findings correlate well with the cranial dis-placement of the early metanephric kidney from its initial location in the pelvic region of the embryo to its final anatomical location at ventral surface of the lumbar vertebrae in most adult species.

The elongation of the vertebral column is more rapid than that of the spinal cord, resulting in the ascent of the spinal cord inside the vertebral canal, causing the conus medullaris to recede cranially. In goat fetuses, the conus medullaris was located between S4 and S5 in group 1, while it shifted to S2 in group 3. In sheep, the conus medullaris recedes cranially from S4 or S3 at 3 months of gestation to S2 in adults (Ghazi and Gholami, 1993a ▶). However, in the human fetus, the conus medullaris shifts from S2-L5 at 4 months to L3-L2 at 7 months of gestation (Nizankowsky and Kurlej, 1982 ▶), whereas in adults, it is located at the lower border of L2 (McCotter, 1916 ▶). In neonate camels, the spinal cord terminates at the middle and caudal thirds of S3 in 80% of the cases, while in 1 year old animals, in 80% of the cases the spinal cord is found to extend between the caudal third of S2 and the cranial third of S3 and in 20% of the cases, it terminates at the middle third of the S2 vertebra (Ghazi et al., 1997). In male dogs, the spinal cord terminates at caudal border of S3 for fetuses of 42 and 52 days of gestation, while in the neonate dog it terminates at the cranial border of S1 (Ghazi et al., 2001b ▶). In cat fetuses, the spinal cord terminates at the cranial third Co_1_ after 37 days of gestation, at the caudal third of S3after 52 days of gestation and at the caudal border of S2 in new born cats, while in the adult cat it terminates at the caudal third of the S2 vertebra (Ghazi et al., 2004b ▶). As for laboratory animals, the termination of the spinal cord in both male and female new born rabbits was at the caudal border of S2, in the male and female new born guinea pigs it was at the caudal border of L6 and in male and female new born rats it was at caudal border of S3 vertebra (Ghazi et al., 2001a ▶). In the Albino mouse this change is from S5 at 1 day of age to L5 at 120 days postnatally (Sakla, 1969 ▶). In the adult east central European hedgehog, the conus is located at the level of T7 vertebra (Malinska et al., 1972 ▶).

It is concluded that the allometric growth rate was highest in the cervical and thoracic regions, followed by the lumbar, sacral and coccygeal regions. Although these differences were significantly different between all regions within each group (P<0.01), no significant difference was found between respective regions of the male and female fetuses. Moreover, in the cervical and thoracic regions of all age groups, the growth rate was isometric, whereas in the lumbar, sacral and coccygeal regions, the growth rate became negatively allometric. This is the main cause of angulation of the spinal nerves and formation of the cauda equina.
